# Evolution of the Intracranial Approaches to Jugular Foramen Tumors: A Surgeon’s Personal Perspective Through Three Illustrative Cases

**DOI:** 10.7759/cureus.530

**Published:** 2016-03-12

**Authors:** Walter C Jean, Daniel R Felbaum

**Affiliations:** 1 Neurosurgery, Medstar Georgetown University Hospital

**Keywords:** jugular foramen, skull base, meningioma, schwannoma, endoscope

## Abstract

Tumors of the jugular foramen remain challenging lesions despite advances in surgical technique and medical technology. Tumors with extensive extra- and intracranial components necessitate both radical neck dissection maneuvers combined with skull base approaches. We present a single surgeon’s perspective in managing these difficult tumors.

## Introduction

Due to its deep location in the skull base with its associated intricate interweaving of neural and vascular structures, the jugular foramen remains very difficult and hazardous to access in modern neurosurgery. The removal of a large tumor of the jugular foramen with significant extracranial component requires a combination of skull base approaches. Most of these techniques involve the mobilization of cranial nerves and ligation of the jugular vein and sigmoid sinus [1]. The risks of cranial nerve palsies and venous insufficiency from these combined extra- and intracranial approaches are high, not to mention that the removal of large amounts of bone near the occipital condyle may have destabilizing effects on the craniocervical junction [1-5].

These surgically complex and highly invasive approaches may therefore be over-aggressive for a Kaye type A jugular foramen tumor, located primarily within the intracranial compartment [4,6]. On the other hand, the retrosigmoid approach, the “work-horse” to access the intracranial portion of the jugular foramen, may be insufficient to remove the intraforaminal portion of the tumor, leading to a high recurrence rate of these challenging tumors [7].

This study involves three patients with intracranial jugular foramen tumors, which illustrates the evolution of a single surgeon’s thought process in approaching these rare tumors. 

## Technical report

Case Presentation

Informed consent was obtained in all patients prior to surgical evaluation and treatment.

Case 1

**Figure 1:**
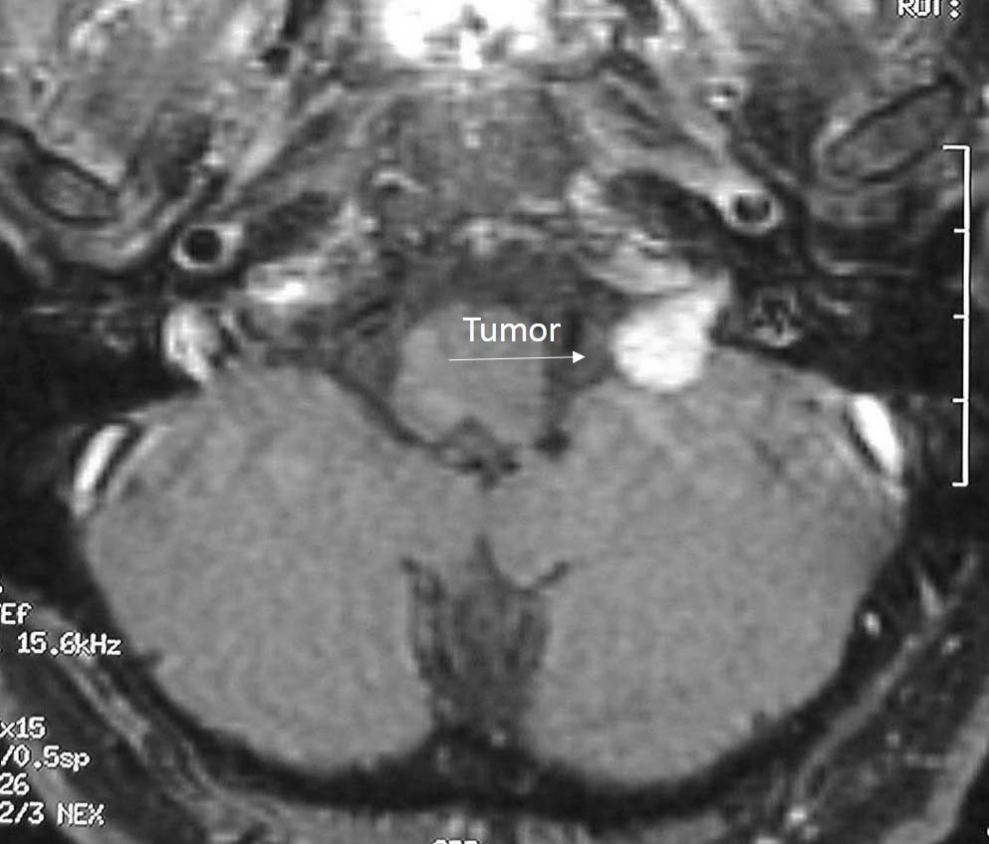
Preoperative MRI of Case 1 Preoperative MRI depicting a homogenously enhancing lesion originating from the jugular foramen.

A 42-year-old male presented with debilitating headaches. A radiographic evaluation revealed an intracranial tumor in the region of the jugular foramen, with extension into the foramen itself (Figure 1).

**Figure 2:**
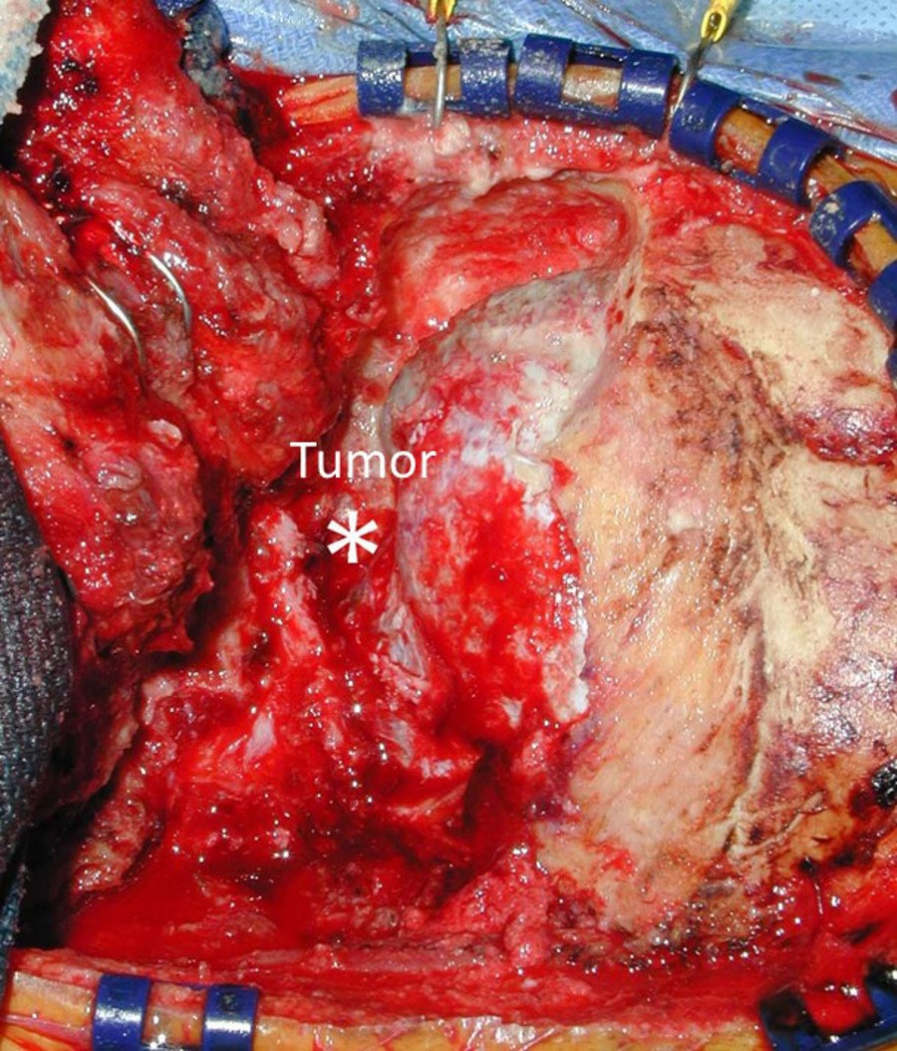
Intraoperative photograph for Case 1 Intraoperative image depicting a far-lateral, retrolabyrinthine, transmastoid, transcondylar approach. The tumor is marked by the asterisk.

**Figure 3:**
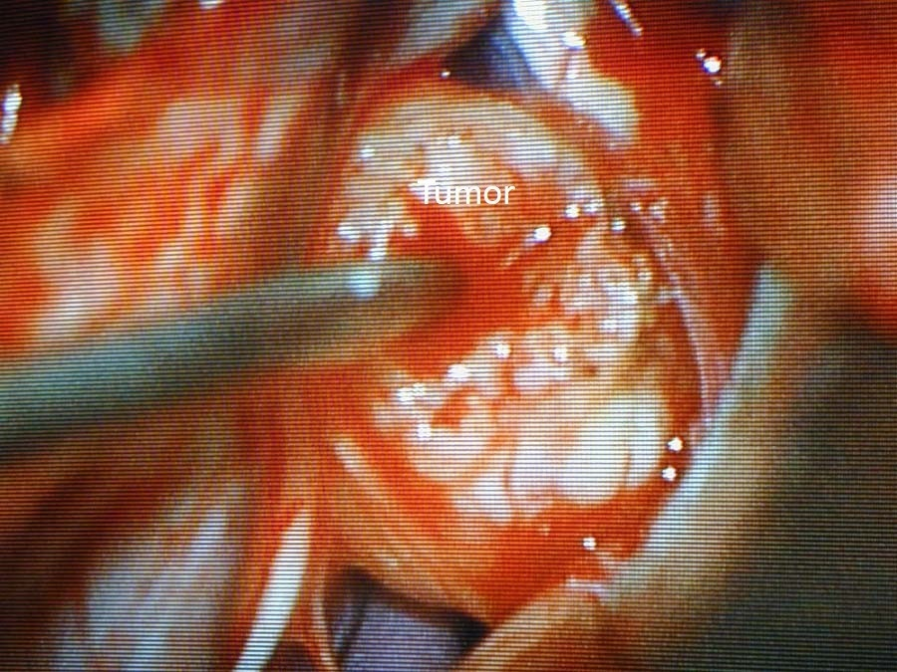
Microscopic photograph of tumor for Case 1 Using a microscope, the lesion is shown in the center of the photograph. The initial dissection of the lesion is begun, with the capsule being coagulated.

A far-lateral, retrolabyrinthine, transmastoid, transcondylar approach was performed to access the tumor (Figure 2 & Figure 3).

**Figure 4:**
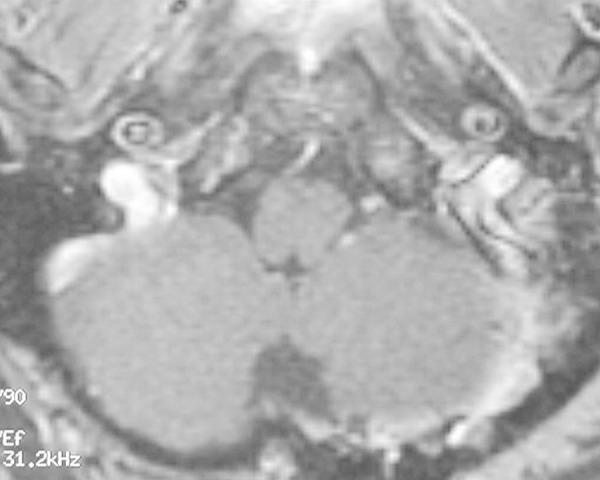
Postoperative MRI of Case 1 Postoperative MRI of Case 1 depicting an apparent total gross resection of the lesion.

Postoperatively, the patient was neurologically intact, with no sign of dysphonia or dysphagia. No tumor residual was seen on the magnetic resonance imaging (MRI) scan (Figure 4).

However, three years later, an intraforaminal regrowth was noted, and the patient was treated with stereotactic radiosurgery to 21 gray (Gy) in three sessions. The patient has been progression free since that time.

Case 2

**Figure 5:**
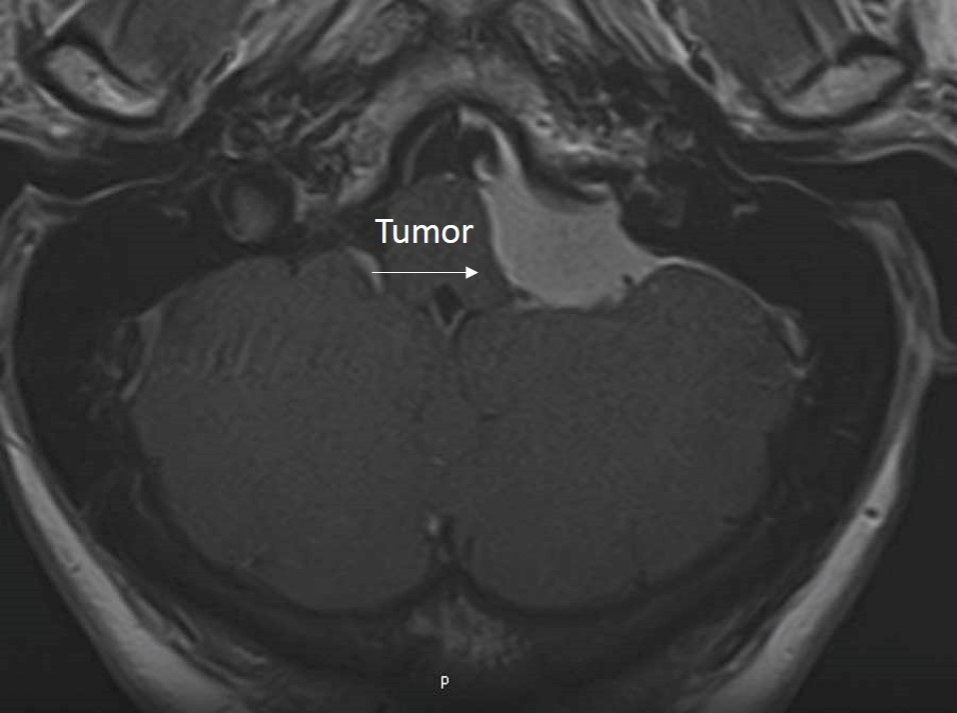
Preoperative MRI of Case 2 Preoperative MRI with contrast depicting a homogenously enhancing lesion with a characteristic dural tail. The lesion is located near the jugular foramen with a component within the cerebellopontine angle.

A 55-year-old female presented with sensorineural hearing loss and tinnitus on the left. An MRI performed showed an enhancing, dural-based tumor centered on the left jugular foramen, impinging on the brainstem (Figure 5).

The tumor extended into the jugular foramen but did not extend into the extracranial space. A far-lateral, transcondylar approach was performed for tumor resection. Intraoperatively, the tumor was noted to engulf all branches of CN IX and X. The tumor was carefully dissected off the brainstem, saving as many branches of the lower cranial nerves as possible. The resection was deemed to be gross total, but the postoperative MRI showed some residual enhancement in the dura near the jugular tubercle.

**Figure 6:**
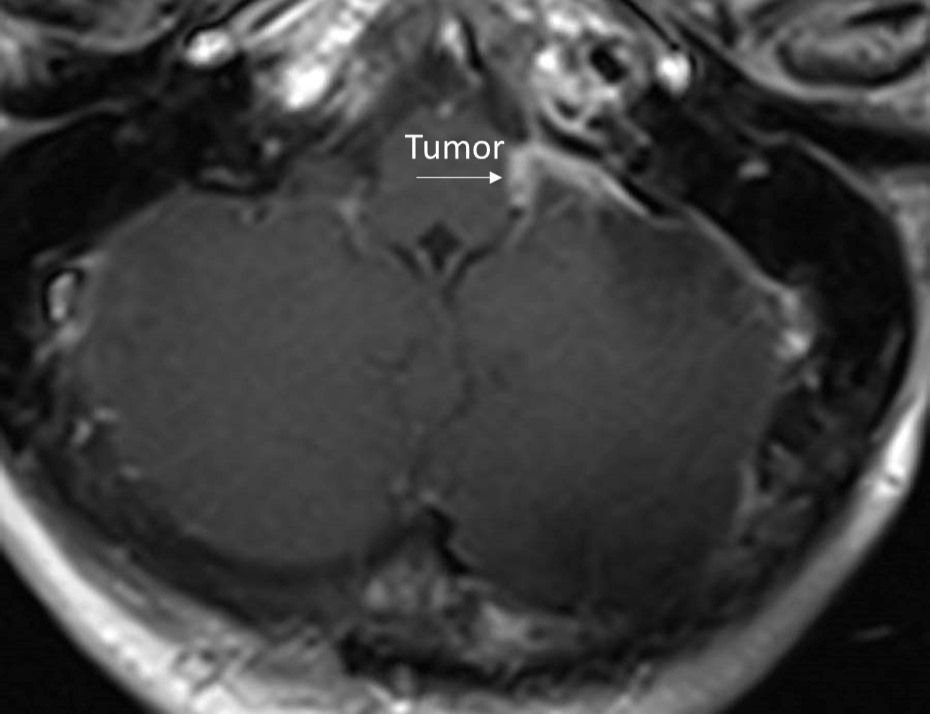
Postoperative MRI of Case 2 Postoperative MRI with contrast depicting a near total gross resection of the jugular foramen lesion via a retrosigmoid approach.

The patient experienced significant dysphagia after the operation and required a percutaneous gastrosotomy tube (PEG) for feeding. Over a prolonged follow-up, the patient’s swallowing function improved and she remains asymptomatic without need of her PEG tube, eating normally. She is being followed with a stable residual tumor on serial imaging (Figure 6).

Case 3

A 39-year-old male presented with severe headaches and pulsatile tinnitus on the right. The MRI showed an egg-shaped tumor, with the narrow portion buried in the right jugular foramen and the broad portion jutting into the cerebellopontine angle. A retrosigmoid approach was performed to resect the tumor. After the cerebellopontine angle component of the tumor was removed, the endoscope was used to visualize the remnant inside the jugular foramen. Under visualization with a combination of the endoscope and microscope, the intraforaminal component was removed completely (Video 1).

**Video 1:** Operative video of a retrosigmoid approach to the jugular foramen Brief illustrative video depicting the preoperative considerations and operative technique employing a retrosigmoid approach to the jugular foramen.

The patient experienced dysphagia and dysphonia after surgery. The latter resolved after 10 days, but the swallowing problem remains at the time of writing. The patient continues to use a gastrostomy for feeding at most recent follow-up.

## Discussion

A wide variety of approaches to the jugular foramen have been described and these can be distilled into three groups: the lateral, anterior, and posterior approaches [7-8]. The anterior group involves the anterior transposition of the facial nerve, osteotomy of the mandible, and drilling of the temporal bone. These approaches are used mainly in combination with other intracranial approaches for jugular foramen tumors with extensive extracranial involvement [7].

Whether starting anterior or posterior to the auricle, the lateral approaches all proceed transtemporal or subtemporal to gain access to the anterolateral aspect of the jugular foramen. All of the approaches in this group require gaining control of the carotid artery and ligating the jugular vein. Some of these approaches, such as Fisch type A, involve the transposition of the facial nerve, thus risking facial paresis [9]. The juxtacondylar approach avoids the risks of hearing loss and facial palsy, but the large amount of bone removed around the atlanto-occipital joint in this approach risks the destabilization of the craniocervical junction [10]. Bulsara et al. recently reported using the Extreme Lateral Inferior Transcondylar Transtubercular Exposure (ELITE) for 15 Kaye type A tumors [3,11]. Although their results were excellent, one must wonder if the risks of the approach were justified when only a small portion of the tumor is in the foramen, with the majority component intradural.

The posterior group is used for tumors located mainly in the cerebellopontine angle with limited extension into the jugular foramen. Although some approaches involve the presigmoid corridor, most use the corridor posterior to the sigmoid sinus [12]. The classic retrosigmoid approach provides great access to the main portion of the tumor in the cerebellopontine angle but extra removal of bone of the occipital condyle, the jugular tubercle and the mastoid may provide better access to the part of the tumor inside the jugular foramen.

Recently, Katsushima et al. described a suprajugular extension of the retrosigmoid approach to gain access to the intraforaminal tumor [2]. The bone above the jugular foramen, in an area bordered by the internal acoustic meatus above and endolymphatic depression laterally, including the intrajugular process and jugular ridge, is removed. Drilling in this tiny area, right on top of delicate cranial nerve branches, is technically challenging, and this technique has yet to been broadly applied in the clinical setting.

Samii et al. applied the endoscope in their vast experience with jugular foramen access. Through a retrosigmoid infralabyrinthine approach, they found that five out of seven patients benefited from endoscopic assistance in aiding tumor resection within the foramen [13]. 

Our experience with these rare and challenging tumors has mirrored the descriptions in the literature, and the evolution of our thought process and surgical technique has progressed with our clinical experience. This is illustrated best by the three cases that are described in this study. Early in our experience, the prevailing idea was that more bone removal translates to better visualization and access of the foramen. We thought this led to better results. However, in case 1, the additional mastoid and petrous bone removal in fact did not significantly enhance the access to the foramen. Furthermore, despite the large amount of bone removed combined with microscopic total resection at the time of surgery, the tumor recurred inside the foramen within three years.

Case 2 illustrated the second “milestone” in our thought evolution. Similar to Sedney et al.’s experience, we began to think that additional bone removal was perhaps unnecessary and, in fact, may be over-aggressive [3]. Thus, we tackled this meningioma mainly with the retrosigmoid approach, adding only the transcondylar drilling. With this approach, we were able to access the jugular foramen adequately. More importantly, five years after her surgery, the patient has remained progression free.

We applied the retrosigmoid approach as a stand-alone in our next “milestone,” illustrated in case 3. After the main portion of the tumor was removed from the cerebellopontine angle, the endoscope was used to visualize and remove the foraminal portion. We found that the endoscope fit through the surgical corridor safely, and provided panoramic visualization of the tumor inside the jugular foramen. Furthermore, while the surgeon held in the endoscope in the non-dominant hand, the dominant hand was able to switch between instruments without difficulty. Although we still used the microscope at the end of the procedure, the endoscope was a critical adjunct. It was able to provide the additional visualization of the jugular foramen, that mastoid and petrous bone removal did not in Case 1. This highlights the marriage of open microsurgical skull base techniques with recent endoscopic advances, as also experienced by other institutions [13].

## Conclusions

Exactly how much bone one needs to eliminate to access a jugular foramen tumor clearly depends on the individual characteristic of each tumor. Early in our careers, the dogmatic belief was that drilling more bone means better results. This has gradually been replaced by a less-invasive stance. This study once again illustrates that endoscopic and microscopic techniques must not be thought of as separate disciplines in skull base surgery, but a continuum of tools, seamlessly interchangeable, equally facile, to be utilized in these challenging procedures. 
